# Combinatorial interaction between two human serotonin transporter gene variable number tandem repeats and their regulation by CTCF

**DOI:** 10.1111/j.1471-4159.2009.06453.x

**Published:** 2010-01

**Authors:** Fahad R Ali, Sylvia A Vasiliou, Kate Haddley, Ursula M Paredes, Julian C Roberts, Fabio Miyajima, Elena Klenova, Vivien J Bubb, John P Quinn

**Affiliations:** * School of Biomedical Sciences, University of LiverpoolLiverpool, UK; § Department of Biological Sciences, University of EssexEssex, UK

**Keywords:** behaviour, CCCTC-binding factor, human serotonin transporter, polymorphism, variable number tandem repeat

## Abstract

Two distinct variable number tandem repeats (VNTRs) within the human serotonin transporter gene (*SLC6A4*) have been implicated as predisposing factors for CNS disorders. The linked polymorphic region in the 5′-promoter exists as short (*s*) and long (*l*) alleles of a 22 or 23 bp elements. The second within intron 2 (Stin2) exists as three variants containing 9, 10 or 12 copies of a 16 or 17 bp element. These VNTRs, individually or in combination, supported differential reporter gene expression in rat neonate prefrontal cortical cultures. The level of reporter gene activity from the dual VNTR constructs indicated combinatorial action between the two domains. Chromatin immunoprecipitation demonstrated that both these VNTR domains can bind the CCCTC-binding factor and this correlated with the ability of exogenously supplied CCCTC-binding factor to modulate the expression supported by these reporter gene constructs. We suggest that the potential for interaction between multiple polymorphic domains should be incorporated into genetic association studies.

*J. Neurochem.* (2010) **112**, 296–306.

Two variable number tandem repeats (VNTRs) in the human serotonin transporter gene, *SLC6A4* (the structure is shown in Fig. 1a) have been implicated in the pathophysiology of many CNS-related disorders (reviewed in Haddley *et al.* 2007). The first identified was a bi-allelic 43 bp insertion/deletion ∼1.2 kb upstream of the transcription start site, termed the *SLC6A4* gene-linked polymorphic region (LPR) (Heils *et al.* 1996; Lesch *et al.* 1996). The most commonly occurring LPR variants contain 14 (short, *s*) or 16 (long, *l*) copies of a 22–23 bp repeat (Fig. 1b) (Heils *et al.* 1997; Nakamura *et al.* 2000). Recent clinical studies indicated that an A/G single nucleotide polymorphism (SNP) in the *l*-allele of the LPR VNTR could affect *SLC6A4* mRNA expression (Hu *et al.* 2005; Wendland *et al.* 2006). The second VNTR found in intron 2 comprises most commonly 9, 10 or 12 copies of a 16–17 bp repeat (Stin2.9, Stin2.10 and Stin2.12 VNTRs respectively) (Battersby *et al.* 1996; Ogilvie *et al.* 1996). We and others have previously demonstrated that these Stin2 VNTRs support differential gene expression *in vitro* (Heils *et al.* 1996; Lesch *et al.* 1996; Lovejoy *et al.* 2003; Klenova *et al.* 2004; Roberts *et al.* 2007) and during neural development in transgenic mice (MacKenzie and Quinn 1999). The potential contribution of genomic variation to direct differential *SLC6A4* gene expression was further supported by the identification of other SNPs in the middle of the first intron; rs16965628 and rs2020933 that showed a significant correlation with the allelic transcript ratio of *SLC6A4*, therefore, contributing to the differential expression of the *SLC6A4* gene (Martin *et al.* 2007).

**Fig. 1:**
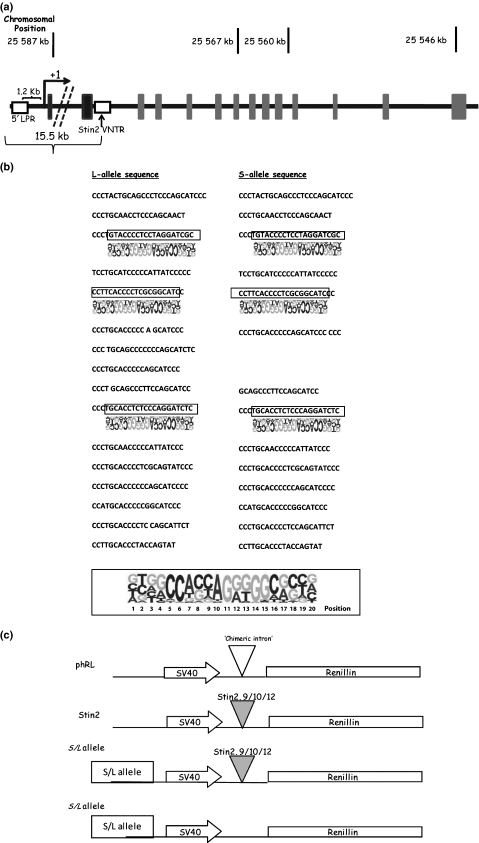
Schematic representation of the *SLC6A4* structure. (a) Schematic representation of the *SLC6A4* structure, highlighting the 5′-LPR and Stin2 VNTRs (white boxes); positioned relative to the transcription start site (indicated as +1). Grey boxes: coding exons and black boxes: non-coding exons. (b) The sequences of the long (*l*) and short (*s*) alleles within the linked polymorphic region (LPR) of the human serotonin transporter gene (*SLC6A4*) are shown on the left and right respectively. Potential CTCF binding sites within the *l*- and *s*-alleles: the best matches on the negative strand of the DNA are boxed based on the CTCF ‘consensus’ identified via a genome-wide screen (Kim *et al.* 2007) which is shown at the bottom of this panel. The size of each base in the consensus is indicative of the probability of its presence at a particular position within the 20 bp motif, with the most restricted variation at 5, 6, 11, 14 and 15. (c) Cartoon illustrating relative positions of the *SLC6A4* VNTRs in the reporter gene constructs (not to scale). The single VNTR *renilla* luciferase reporter constructs have either the *s*- or *l*-allele alone upstream of the minimal SV40 promoter, or one of the intron 2 VNTR alleles (either Stin2.9, Stin2.10 or Stin2.12) located alone within a synthetic intron of the phRL vector upstream of the *renilla* coding sequences. The dual constructs were generated by cloning a combination of one of the 5′-LPR alleles with one of the Stin2 VNTR alleles in the same positions, respectively, as used in the single reporter constructs.

Many studies have linked these VNTRs with disorders including increased risk of developing depression (Cervilla *et al.* 2006), bipolar depression (Cho *et al.* 2005), anxiety (Lesch *et al.* 1996; Hariri *et al.* 2002), predisposition to suicide or depression following stressful life events (Caspi *et al.* 2003; Zalsman *et al.* 2006) and a predisposition to obsessive compulsive disorder (Bengel *et al.* 1999). However, these findings were not always reproducible in independent studies (Rees *et al.* 1997; Hoehe *et al.* 1998). Various meta-analyses suggested no significant allelic frequency association of the Stin2 VNTRs with affective disorders but they did favour an association of the LPR VNTR with these disorders (Cho *et al.* 2005; Lasky-Su *et al.* 2005).

We have previously shown that the transcription CCCTC-binding factor (CTCF) is a regulator of Stin2 VNTR function (Klenova *et al.* 2004; Roberts *et al.* 2007). CTCF is a DNA-binding protein with a role in transcriptional activation or silencing in a context-dependent fashion and may also act as an insulator in epigenetic remodelling (Ohlsson *et al.* 2001; Klenova *et al.* 2002). There are several potential high affinity CTCF binding sites present in the LPR (Fig. 1b) and we hypothesised that, as transcriptional domains, the LPR VNTR and Stin2 VNTR can act in concert to modulate *SLC6A4* gene expression, and that CTCF may be one factor to coordinate such activity. To support such a hypothesis we have addressed the ability of the VNTRs to function together in modulating reporter gene activity in neuronal cultures and assessed if such function was regulated by CTCF.

## Experimental procedures

Comprehensive details of the material and methods are given online in Appendix S1.

### Generation of reporter gene constructs

The promoter LPR and Stin2 VNTR fragments were amplified by PCR using the primers: forward 5′-GGGGTACCCCTGGCGTTGCCGCTCTGAATGC-3′ and reverse 5′-CCGCTCGAGCGGAGGGACTGAGCTGGACAACCAC-3′ and forward 5′-ATGGCGCGCCGGTACCTCACAGGCTGCGAGTAGA-3′ and reverse 5′-AACGGCGCGCCTCGAGTGGCCTCTCAAGAGGA-3′ respectively. VNTR fragments were cloned into a modified *renilla* luciferase reporter vector [modified phRLSV40 (Guindalini *et al.* 2006)], such that each individual promoter LPR fragment was placed upstream of a minimal SV40 promoter (creating phRL_long and phRL_short) and each individual Stin2 VNTR fragment was placed within a synthetic intron located upstream of the *renilla* luciferase coding sequences (creating phRL_Stin2.9, phRL_Stin2.10 and phRL_Stin2.12). Dual constructs termed: L9, L10, L12, S9, S10 and S12 were created such that combinations of alleles from the two VNTRs were present at their respective positions within the plasmid (Fig. 1c).

### Reverse transcription PCR

Total RNA was extracted from rat prefrontal cortical cells with Trizol reagent (Invitrogen, Paisley, UK) and reverse-transcribed to cDNA using Reverse Transcription System (Promega, Southampton, UK) following manufacturer’s instructions. PCR was performed to confirm *SLC6A4* mRNA expression using the primers forward 5′-TTCCTCCTGTCCGTCATTGG-3′ and reverse 5′-GGTGGATCTGCAGGACATGG-3′ from exons 1 and 4 respectively.

### Primary cell culture, transient transfections and luciferase assays

Male Wistar albino rats (2 to 7-day old) were used to generate primary cortical cultures. All animals were culled under local and national schedule one guidelines. Reporter constructs (1 μg) or modified phRLSV40 (1 μg) and modified pMLuc-2 (expressing *Firefly* luciferase as an internal control) were co-transfected using ExGen500 *in vitro* transfection reagent following manufacturer’s guidelines (Fermentas, York, UK). For co-transfections with CTCF, cells were transfected with 1 μg of reporter gene and 1 μg of expression vector pCI-CTCF (Klenova *et al.* 2004), or to standardise total DNA concentration, pGL3basic (Promega). Cells were harvested and assayed using the Dual Luciferase Reporter Assay System (Promega) and luminescence was measured using a Glomax 96 microplate luminometer (Promega). Mean and SEM were calculated from the results of three independent experiments performed in triplicate.

### Statistical analysis

Data were subjected to statistical analysis using one-way anova and a significance level of 0.05. The Dunnett’s (two-sided) *t* method was used as a *post hoc* test to account for multiple comparisons.

### Chromatin immunoprecipitation

DNA and protein from 10^7^ JAr cells (human placental choriocarcinoma) were cross-linked with 1% formaldehyde and mixed thoroughly at 20°C for 10 min. Chromatin immunoprecipitation (ChIP) was performed using ChIP-IT express (Active Motif, Rixensart, Belgium) (see Appendix S1) and the antibodies: mouse monoclonal anti-human CTCF (BD Transduction Laboratories, Lexington, KY, USA) and polyclonal rabbit anti-mouse IgG, as a control (Abcam, Cambridge, UK). PCR primers used for LPR amplification were forward 5′-GGGGTACCCCTGGCGTTGCCGCTCTGAATGC-3′ and reverse 5′-CCGCTCGAGCGGAGGGACTGAGCTGGACAACCAC-3′.

### Electrophorectic mobility shift assays

The human recombinant CTCF protein was purified from baculovirus (Chernukhin *et al.* 2000). An oligonucleotide pair, 5′-TCGACCCCTCGCAGTATCCCCCCTGCA-3′ (100 ng) designed as overhanging complementary strands, was annealed then labelled with α-^32^P dATP (specific activity 6000 Ci/mM) (Amersham, Buckinghamshire, UK) using DNA Polymerase I, Large (Klenow) Fragment (NEB, Hitchin, Hertfordshire, UK). Electrophoretic mobility shift analysis (EMSA) assays were performed as previously described (Klenova *et al.* 2004).

## Results

We have previously addressed the expression of the intronic Stin2 VNTR cloned upstream of a minimal promoter in standard reporter gene constructs (Klenova *et al.* 2004; Roberts *et al.* 2007). The limitation of these studies was that such vectors did not properly reflect the genome context. To partially address this issue, we placed the *SLC6A4* LPR VNTR and Stin2 VNTR variants in more appropriate locations within the reporter vector. Specifically the Stin2 alleles were cloned into an intronic site of the phRL reporter vector and the LPR VNTR variants were cloned upstream of the SV40 promoter (Fig. 1c). Although this placed the Stin2 VNTR within an intron mimicking the *in vivo* position, it is important to acknowledge that these two domains are ∼15.5 kb apart in the human genome.

**Fig. 2:**
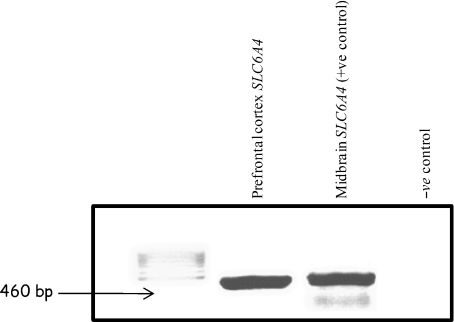
Agarose gel showing the reverse transcription PCR products of the *SLC6A4* gene amplified from sections of neonatal Wistar rat brain. Lane 1: Mass ruler DNA Ladder; lane 2: *SLC6A4* RT-PCR amplification from rat prefrontal cortex, amplifying a 460 bp cDNA fragment of the expected size; lane 3: *SLC6A4* RT-PCR amplification from rat midbrain; lane 4: negative control (water). Sizes of the amplified fragments are indicated.

We had previously modified the phRL vector by introduction of a unique enzyme site into the intron and utilised this to address the function of intronic dopamine transporter VNTR variants (Guindalini *et al.* 2006). We also generated constructs using the same backbone but with only the LPR or Stin2 variants cloned in their respective locations. This allowed us to address not only the hypothesis that these two *SLC6A4* VNTR domains could act *in cis* but how combined effects may differ from each independent domain alone, and if interactions or synergisms were possible between these VNTR domains that involved *trans* factors. Recently, an A/G SNP in the *l*-allele of the LPR VNTR was reported to affect mRNA expression levels of *SLC6A4* gene (Hu *et al.* 2005; Wendland *et al.* 2006), we therefore determined by sequence analysis that the SNP in our clone was ‘A’. Functional analysis was performed in primary cultures of rat prefrontal cortex (PFC) primary culture, which were enriched for neurons, because it has been reported that these cells express the endogenous *SLC6A4* gene (Fumagalli *et al.* 1996; Hansson *et al.* 1998; Lebrand *et al.* 1998). However, because of the mixed cell population of the PFC, a proportion of non-neuronal glial cells are present even though cells were prepared on poly-d-lysine coated plates to increase the proportion of neuronal cells. Expression of the *SLC6A4* mRNA in the rat PFC has been reported to peak early in postnatal development at day 10 and gradually decrease to weak expression at day 28, with no expression been recorded in adult rats (Fumagalli *et al.* 1996; Hansson *et al.* 1998; Lebrand *et al.* 1998). We confirmed *SLC6A4* mRNA expression in our primary cultures by RT-PCR amplifying a 460 bp cDNA fragment with primers in exons 1 and 4, to eliminate the possibility of product amplified from contaminating DNA (Fig. 2). The cDNA of midbrain sections was included in the RT-PCR as a positive control for *SLC6A4* expression.

### CTCF can bind to and regulate expression of both the LPR and Stin2 alleles

We have previously demonstrated that the transcription factor CTCF binds and differentially regulates the Stin2 VNTRs in clonal cell lines (Klenova *et al.* 2004; Roberts *et al.* 2007). In this study, we assessed the ability of the individual LPR and Stin2 VNTRs to support reporter gene expression in rat prefrontal cortical neuronal cells (PFC).

**Fig. 3:**
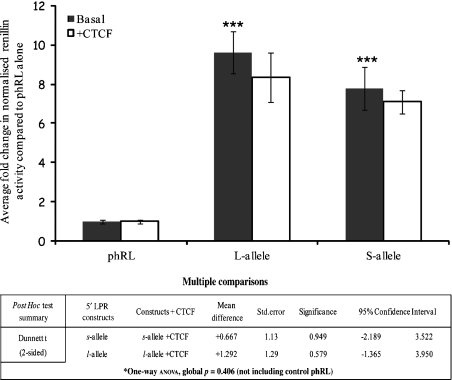
The *SLC6A4* 5′-LPR alleles support similar levels of activity in rat prefrontal cortical cells and CTCF does not regulate the activity of either allele. Rat prefrontal cortical cells were transfected as described in methods with 1 μg of reporter gene construct plus either 1 μg of CTCF expression vector or 1 μg of pGL3b, to standardise for total DNA concentration; 20 ng of pMLuc-2 *Firefly* luciferase was used as internal control. Bars represent the fold change in normalised renillin activity supported by the VNTR compared with the phRL control under basal conditions (black bars) or following CTCF over-expression (white bars). Transfections were performed in triplicates in at least five independent experiments and the mean normalised renillin values were calculated together with the SEM represented by error bars. One-way anova using Dunnett’s *t* (two-sided) as a *post hoc* test indicated that both 5′-LPR (*l*- and *s*-) alleles displayed a significant up-regulation compared with the phRL control (∼10 and 8-fold, respectively, ***p ≤ 0.001), whereas no significant difference was observed when the two alleles were compared with each other (*p* = 0.25, SE 1.018). Neither allele was significantly affected by CTCF over-expression.

**Fig. 4:**
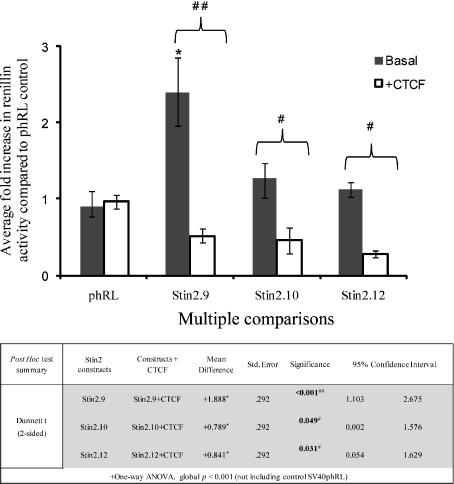
CTCF significantly repressed the expression of the three allelic variants of the Stin2 reporter construct in rat prefrontal cortical cells. Rat prefrontal cortical cells were transfected as described in methods with 1 μg of reporter gene construct plus either 1 μg of CTCF expression vector or 1 μg of pGL3b, to standardise for total DNA concentration; 20 ng of pMLuc-2 *Firefly* luciferase was used as internal control. Bars represent the fold change in normalised renillin activity supported by the VNTR compared with the phRL control under basal conditions (black bars) or following CTCF over-expression (white bars). Transfections were performed in triplicates in at least five independent experiments and the mean normalised renillin values were calculated together with the SEM represented by error bars. One-way anova using Dunnett’s *t* (two-sided) as a *post hoc* test indicated that Stin2.10 and Stin2.12 supported no additional activity over the phRL control (*p* = 0.814 and 0.981 respectively), whilst Stin2.9 supported reporter gene expression, ∼2.5-fold higher than the phRL control (*p ≤ 0.001). Stin2.9 also showed significant change in activity compared with both Stin2.10 and Stin2.12 reporter constructs *p* ≤ 0.001. Stin2 variants were significantly repressed by CTCF over-expression (^#^*p* ≤ 0.05 and ^##^*p* ≤ 0.001).

In this cell system, the *l*- and *s*-LPR alleles demonstrated 10- and 8-fold activation, respectively, over the phRLSV40 control (Fig. 3). In contrast, the Stin2.10 and Stin2.12 (the most common intronic variants) only weakly supported gene expression, over the phRLSV40 control (Fig. 4). However, the rare nine copy allele, supported gene expression, showing 2.5-fold activation in comparison with the control, phRLSV40 (Fig. 4).

**Fig. 5:**
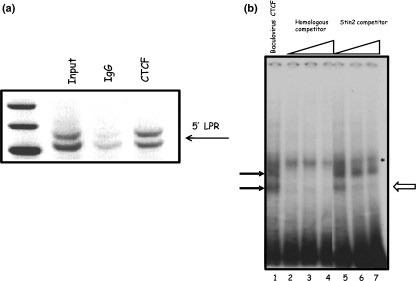
CTCF binds the 5′-LPR VNTR of the *SLC6A4* gene. (a) ChIP analysis of the *in vivo* interaction of CTCF with the 5′-LPR alleles. ChIP analysis was performed as described in the methods section. IgG was included as a control for non-specific background binding. CTCF was found to bind both *l*- and *s*-alleles of the 5′-LPR (upper and lower bands respectively). (b) EMSA analysis of CTCF binding to a repeat element of the 5′-LPR. EMSA analyses were performed as described in the methods section. CTCF purified from Baculovirus showed that two specific protein/DNA complexes could be observed (b, lane 1, closed arrows), which could be competed with homologous competitor (b, lanes 2–4). Competitors were added from 10- to 100-fold molar excess. However, only one of these complexes was competed with the Stin2 oligonucleotide competitor (b, lanes 5–7, open arrow). The complex indicated by asterisk possibly represents a non-specific DNA–protein complex, because it did not compete with the specific competitor.

Based on the CTCF ‘consensus’ motif, we identified several potential CTCF binding sites within the LPR (Fig. 1b). To confirm CTCF binding to this region, we performed ChIP in human JAr cells, as rodents do not possess the same *SLC6A4* polymorphism. In JAr cells, which express the endogenous *SLC6A4* gene and are heterozygous for LPR alleles and contain the 10 and 12 copy variants of the Stin2 VNTR, enrichment for CTCF binding was observed for both alleles of the LPR, compared with the IgG control (Fig. 5a).

To further confirm the binding of CTCF to the 5′-LPR we performed EMSA using baculovirus purified CTCF (Fig. 5b). The 5′-LPR domain is much greater than the size usually preferred for EMSA, thus we used an oligonucleotide which corresponded to a repeat element of the VNTR sequence that contained a CTCF binding motif. Two specific complexes were observed, these were competed with homologous competitor (Fig. 5b). Interestingly, only one of these complexes was effectively competed by an oligonucleotide sequence corresponding to one of the Stin2 VNTR repeat elements, suggesting different affinities for CTCF binding.

We complemented the binding studies by addressing the ability of the exogenous CTCF expressed from an expression construct to modulate expression of either the LPR or Stin2 reporter gene cassettes. Co-expression of CTCF did not significantly affect the expression supported by either of the LPR variants (Fig. 3) but resulted in robust repression of all three Stin2 variants (Fig. 4). The reporter cassette (phRLSV40) without either VNTR, was unaffected by CTCF.

### The dual LPR and Stin2 alleles support differential reporter gene expression *in cis*

**Fig. 6:**
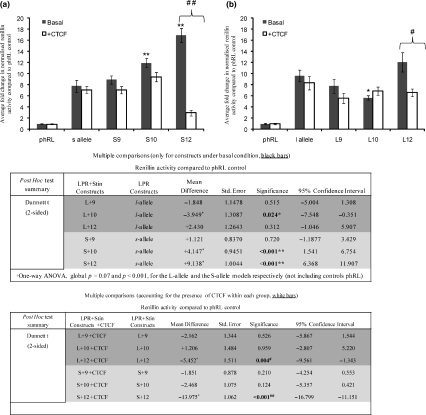
The *SCL6A4* VNTR dual constructs support differential levels of reporter gene expression and CTCF differentially regulates the expression of these dual constructs in rat prefrontal cortical cells. Rat prefrontal cortical cells were transfected as described in methods with 1 μg of reporter gene construct plus either 1 μg of CTCF expression vector or 1 μg of pGL3b, to standardise for total DNA concentration; 20 ng of pMLuc-2 *Firefly* luciferase was used as internal control. The *renilla* luciferase dual reporter constructs generated were a combination of either of the 5′-LPR alleles cloned upstream of the minimal SV40 promoter with either of the Stin2 VNTR alleles located within a synthetic intron of the phRL vector. Bars represent the fold change in normalised renillin activity supported by the VNTR compared with the phRL control under basal conditions (black bars) or following CTCF over-expression (white bars). Transfections were performed in triplicates in at least three independent experiments and the mean normalised renillin values were calculated together with the SEM represented by error bars. One-way anova using Dunnett’s *t* (two-sided) as a *post hoc* test indicated that (a) Both S10 and S12 were capable of directing significantly higher activity compared with *s* alone (*p* < 0.001 for both), whereas S9 did not display significant difference compared with the *s* construct alone (*p* = 0.72) *p ≤ 0.05 and **p ≤ 0.001. (b) L10 showed a significant decrease in reporter gene activity compared with *l*-alone (*p* = 0.024), whilst neither L9 nor L12 showed significant change in activity compared with *l*-alone (*p* = 0.515 and 0.312 respectively). CTCF displayed differential activity on the dual reporter constructs by significantly repressing the activity of (a) S12 and (b) L12 (^##^*p* ≤ 0.001 and ^#^*p* ≤ 0.05), whilst no significant effect was observed with the other constructs as illustrated by the *post hoc* test applied.

We hypothesised that as both VNTRs in the promoter and intron 2 share at least one signal transduction pathway which would be regulated by CTCF this might result in differential reporter gene expression directed by distinct combinations of the VNTRs. We therefore analysed activity supported by the dual constructs containing either the *l*- or *s*-allele in a promoter position and a Stin2 VNTR located in the intron of the reporter plasmid. These constructs demonstrated a number of distinct expression profiles based on the genotype of the VNTRs. Strikingly, the Stin2.10 and Stin2.12 variants which did not support additional activity compared with the phRLSV40 alone, when in conjunction with the *s*-variant (S10 and S12) were capable of directing higher activity compared with *s*-alone (*p* < 0.001) (Fig. 6a). However, the Stin2.9 in combination with the LPR *s*-variant (S9), displayed a similar pattern of expression to *s*-alone. These data suggested a combinatorial rather than an additive mechanism of gene regulation by the two VNTRs.

A complex expression pattern from the reporter was also obtained when the Stin.2 variants were combined with the LPR *l*-allele (Fig. 6b). The Dunnett’s *post hoc* test suggested that L10 showed a significant decrease in reporter gene activity compared with *l*-alone, whilst neither L9 nor L12 showed significant changes in activity compared with *l*-alone. Our analysis also indicated that L12 significantly differed from both L10 and L9, reinforcing that the combinatorial variation cannot be predicted from the individual VNTR activity in this model.

We then tested the effects of CTCF on these constructs. The Stin2 VNTR variants alone were all repressed by over-expression of CTCF (Fig. 4), whilst CTCF had no effect on the LPR constructs (Fig. 3). We therefore addressed the potential regulation of the dual VNTR reporter gene constructs by CTCF. In response to CTCF, the dual constructs showed a variety of responses (Fig. 6). Specifically, over-expression of CTCF significantly repressed the activity of L12 and S12 whilst no significant effect was observed with other dual constructs (Fig. 6). This would suggest a distinct response of Stin2.12 irrelevant of the LPR allele present. The perceived decrease in L10 over *l*-alone, did not reach significance in the presence of CTCF.

## Discussion

Several groups including our own have demonstrated differential effects of the *SLC6A4* LPR and Stin2 VNTRs on the expression of reporter genes in cell lines. Further, the function of these VNTRs to act as a repressor or enhancer was cell type and promoter context dependent; this most likely reflected the different complement of transcription factors in these cells that could regulate the domains (Heils *et al.* 1996; Lesch *et al.* 1996; Mortensen *et al.* 1999; Sakai *et al.* 2002; Lovejoy *et al.* 2003; Klenova *et al.* 2004; Roberts *et al.* 2007). Both these VNTRs have been clinically determined as predisposing factors for various psychiatric disorders including unipolar and bipolar depression (Cho *et al.* 2005; Cervilla *et al.* 2006), anxiety (Lesch *et al.* 1996; Hariri *et al.* 2002) and predisposition to obsessive compulsive disorder (Bengel *et al.* 1999). However, these domains have also been disputed as susceptibility factors and meta-analysis has raised further questions (Cho *et al.* 2005; Lasky-Su *et al.* 2005).

To the authors’ knowledge, with the exception of Kaiser *et al.* (2002) and Hranilovic *et al.* (2004) all studies in the literature have focused on the effect of only one of the *SLC6A4* VNTRs, either LPR or Stin2, on expression of the *SLC6A4* and association with affective disorders (Lesch *et al.* 1996; Bengel *et al.* 1999; Hariri *et al.* 2002; Cho *et al.* 2005). Here we demonstrate, in rat neuronal cells, which express the endogenous *SLC6A4* gene that the 5′-LPR and Stin2 VNTRs within the *SLC6A4* gene, are at least in part, are likely to be on the same signalling pathway regulated by CTCF. We further demonstrate that the 5′-LPR and Stin2 VNTRs can support differential gene expression when analysed in concert using constructs designed to mimic their endogenous positions in the gene, albeit as indicated previously these two domains are 15.5 kb apart in the human genome. However, CTCF is known to mediate looping between distant DNA elements, therefore the mechanistic basis of regulation by CTCF *in vivo* and in the reporter system used may be similar and rely on loop formation between two CTCF sites (Phillips and Corces 2009; Zlatanova and Caiafa 2009). However as the functional outcomes from the dual constructs containing *l*- and *s*-variants are different (Fig. 6), the properties of such hypothetical loops would be expected to vary. One possible scenario would be that CTCF may utilise different combinations of its zinc fingers at different binding sites thus releasing different surfaces for interaction with protein partners (Ohlsson *et al.* 2001). This, in turn, may result in the formation of distinct functional high order DNA–protein complexes leading to different functional outcomes (Wallace and Felsenfeld 2007). This will need to be investigated further.

Although the VNTRs are likely to function in the adult CNS in response to challenge we have previously demonstrated that in a transgenic mouse model the Stin2 variants supported differential reporter gene expression during development in the CNS region initially involved in serotonergic lineage (MacKenzie and Quinn 1999). Furthermore, there are also increasing suggestions that the role of the *SLC6A4* polymorphisms may be more pronounced during embryogenesis and development, thus raising the possibility that disrupting normal maturation of certain neuronal networks critical for normal adult functions, will influence behaviour and increase vulnerability to psychiatric disorders in adults (Caspi *et al.* 2003; Ansorge *et al.* 2004; Parsey *et al.* 2006). This might be relevant for our current study as we utilised rat neonate prefrontal cortical cells which *in vivo* demonstrate differential *SLC6A4* expression in the rat in early postnatal period (Fumagalli *et al.* 1996; Hansson *et al.* 1998; Lebrand *et al.* 1998), therefore the functional consequence of these VNTR polymorphisms could to be support differential expression of the *SLC6A4* gene at this point based on genotype.

When the LPR variants were analysed individually, there was no differential activity between the two variants, *l*- and *s*-, on reporter gene expression levels (Fig. 3). This agrees with previous studies in COS-7, PC-12 and raphe-nucleus-derived RN46A cells which found that these VNTR variants did not support differential expression (Willeit *et al.* 2001; Kaiser *et al.* 2002; Sakai *et al.* 2002). Others, however, have demonstrated that these alleles did support differential activity (Heils *et al.* 1996; Lesch *et al.* 1996). Similar conflicting data exist based on mRNA analyses (Lesch *et al.* 1996; Mortensen *et al.* 1999; Hranilovic *et al.* 2004; Lim *et al.* 2006). Therefore, studies on the abilities of the LPR VNTR variants to support differential gene expression, like the clinical correlations themselves do not agree. These contradictory reports may arise from differences in experimental parameters (reviewed in Haddley *et al.* 2007) or demonstrate tissue-specific factors or variation in the concentration of specific transcription factors in a particular cell type. The latter is consistent with our data on manipulation of CTCF concentration altering reporter gene expression supported by the Stin2 VNTR in human embryonic kidney cells, COS-7 and JAr cells (Klenova *et al.* 2004; Roberts *et al.* 2007). Indeed in this study, CTCF repressed the activity driven by the Stin2 variants in the neuronal cultures, when cloned within the synthetic intron construct, which contrasted with our previous data in non-neuronal cell lines in which CTCF increased activity from the Stin2 variants when cloned upstream of the SV40 promoter (Klenova *et al.* 2004; Roberts *et al.* 2007). Without rigorous testing of all combinations and manipulation of transcription factor concentration we can however state that CTCF concentration will modulate VNTR function.

Many previous studies have associated one or other of these individual *SLC6A4* VNTRs with affective disorders (Lesch *et al.* 1996; Bengel *et al.* 1999; Hariri *et al.* 2002; Cho *et al.* 2005). We have now demonstrated that these two VNTRs from the *SLC6A4* gene, in the context of the same reporter system, support differential gene expression based on copy number of both VNTRs. Furthermore, the capability of the dual VNTR constructs to support differential gene expression, under basal growth conditions and in response to over-expression of CTCF, would suggest they act in a manner which is simply not additive from the action of the individual construct in the same cell. For example, the LPR *s*-allele supported an approximate eightfold increase over the phRLSV40 control (Fig. 3) whilst the Stin2.12 allele did not support additional activity over the control when constructs were tested individually (Fig. 4). However, when combined in the dual construct, a 17-fold increase over phRLSV40 was observed (Fig. 6). These analyses may explain, in part, some of the contradictory reports in the literature and indicate that the abilities of both of the VNTRs in the *SLC6A4* gene to act *in cis* should be considered when addressing their correlation to specific neurological disorders, furthermore the combined genotype of an allele may mediate a response to a particular drug or stimulus. As both domains are transcriptional regulators this easily allows for a gene–environment parameter to be factored into a correlation of the VNTRs with a specific condition. It is possible that the impact of the genotype only becomes apparent following exposure to environmental factors, or in response to a particular stimulus e.g., stresses or drugs (Murphy *et al.* 2001). For example, various studies have demonstrated that carriers of the *s*-allele of the 5′-LPR are more likely to develop an episode of major depression only following stressful life events (Bennett *et al.* 2002; Champoux *et al.* 2002; Barr *et al.* 2003; Caspi *et al.* 2003). Clearly such events could alter the transcription factor complement in the cell thus altering VNTR mediated expression; this could be significant both in the adult and during development. Therefore, challenges altering the function of these VNTRs could ultimately influence an individual’s vulnerability to environmental stress and tendency to develop psychiatric disorders by altering both the level of expression of the *SLC6A4* gene or perhaps event the tissue-specific expression during development. Such parameters should be taken into consideration in the design of psychiatric genetics and association studies (Caspi *et al.* 2003; Parsey *et al.* 2006). Our data on the combinatorial potential of the VNTRs is consistent with analysis of *SCL6A4* expression in lymphoblast cell lines which failed to find a correlation with the LPR VNTR, but did find evidence for a combined effect of LPR and Stin2 VNTRs (Hranilovic *et al.* 2004). These data may encourage a better appreciation of how different variants could function in concert *in vivo* to allow better statistical correlations of these domains with a particular disorder by addressing multiple variants on the same allele. Indeed for *SLC6A4* there may be other promoter variants that modulate the activity of the VNTRs.

An important point from our analysis is perhaps not the absolute levels of reporter gene observed, nor indeed whether they are repressed or activated by over-expression of CTCF, but rather that the potential for differential gene expression supported by combinations of the specific variants exists. The action of CTCF highlights the potential role for epigenetic modulation of expression of *SLC6A4* gene via these VNTRs, which could result in long-term differences in gene expression. Recently, Philibert *et al.* (2007) suggested that mechanisms linking the *SLC6A4* polymorphisms vulnerability to epigenetic effects should be considered in association studies, as they demonstrated methylation of a CpG island in the 5′-region of the *SLC6A4* significantly affects expression of the *SLC6A4* gene and that the extent of that effect is dependent on the 5′-LPR genotype. In this respect, the action of CTCF to modulate the activity of CpG domains might become relevant to future studies.

## Abbreviations used:

ChIP: chromatin immunoprecipitation
CTCF: CCCTC-binding factor
EMSA: electrophoretic mobility shift analysis
LPR: linked polymorphic region
PFC: prefrontal cortex
SNP: single nucleotide polymorphism
Stin2: second VNTR in intron 2
VNTR: variable number tandem repeat

## Supporting information

**Appendix S1** Experimental procedures.

As a service to our authors and readers, this journal provides supporting information supplied by the authors. Such materials are peer-reviewed and may be re-organized for online delivery, but are not copy-edited or typeset. Technical support issues arising from supporting information (other than missing files) should be addressed to the authors.
