# Supplementary material for: Combinatorial interaction between two human serotonin transporter gene variable number tandem repeats and their regulation by CTCF
Source: J Neurochem. 2010 Jan;112(1):296–306. doi: 10.1111/j.1471-4159.2009.06453.x (PMC2848977; doi:10.1111/j.1471-4159.2009.06453.x)
Supplement: Supplementary file 1 [file jnc0112-0296-SD1.doc]

**Experimental Procedures**

**PCR amplification**

The promoter LPR VNTR was amplified by polymerase chain reaction (PCR) using the primers: forward: 5’-**ggggtacccct**ggcgttgccgctctgaatgc-3’ and reverse 5’- **ccgctcgagcgg**agggactgagctggacaaccac-3’ (modified from Lesch *et al.*, 1997). Restriction sites for *XhoI* and *Acc65I* were introduced (indicated in bold) at either end of the product to aid directional cloning in a variety of vectors. The Stin2 VNTR was amplified using the primers: forward 5’-**atggcgcgccggtacc**tcacaggctgcgagtaga-3’ and reverse 5’- **aacggcgcgcctcga**gtggcctctcaagagga-3’. A restriction site for *AscI* was introduced for subsequent cloning, and *KpnI* and *XhoI* restriction sites were introduced to aid orientation. PCR reaction mixes contained 100 ng of DNA template, 0.1 μM of each primer, 2.5 units Diamond DNA polymerase (Bioline), 1x Diamond polymerase buffer (Bioline), 0.2 mM dNTPs, 2 mM MgCl_2_, 1 M betaine and water to a final volume of 50 μl per reaction. Prior to addition of PCR mix, the JAr cell DNA template, which is heterozygous for the 5’ LPR and the intronic VNTR (Stin 2.10 and Stin 2.12) alleles, was denatured for 1 min at 99 ^o^C. PCR was performed for 35 cycles: 95 °C denaturing (1 min), 65 °C annealing (1 min), 72 °C extension (1 min), using a Px2 thermal cycler (Thermo Scientific). The Stin2.9 allelic variant was gifted by Dr. Gerome Breen, Institute of Psychiatry, King’s College London.

**LPR VNTR cloning into *Firefly* luciferase reporter vector**

The LPR VNTR fragments amplified by PCR were cloned into the intermediate vector pT7_Blue (Novagen) via blunt-end cloning at the *EcoRV* site. Positive clones were confirmed by sequencing and VNTR inserts released by enzymatic digestion with *XbaI* and *BamHI*. Subsequently, VNTR fragments were cloned into *NheI* and *BglII* sites in the multiple cloning site of the pGL3p vector (Promega) which carries a reporter gene (*Firefly* luciferase driven by a minimal SV40 promoter) creating pGL3p_long and pGL3p_short.

**Modification of the phRL luciferase vector**

A renillin expression vector, phRL_null that contained a synthetic intron (Promega) was modified for subsequent cloning (Guindalini *et al*., 2006). The SV40 promoter was removed from pGL3p with *NotI* and *NcoI*, blunted and subsequently cloned into the *EcoICRI* site of the multiple cloning site of phRL_null. An *AscI* linker was inserted into the intron of this plasmid at the *BbsI* site to allow subsequent insertion of the Stin2 allele cassette. This plasmid was termed modified phRLSV40.

**LPR VNTR cloning in renillin luciferase reporter vector**

The LPR constructs (pGL3p_long and pGL3p_short) were digested with *BamHI* and *HindIII* extracting the fragment containing the LPR VNTR, the SV40 promoter and the β-lactamase gene. This was subsequently ligated to a *BamHI* and *HindIII* fragment from the modified phRLSV40 vector (containing a synthetic intron, the renillin reporter gene and a SV40 polyA) generating phRL_long and phRL_short.

**Stin 2 VNTR and dual construct cloning**

The Stin2 VNTR PCR products were cloned into the intronic *AscI* site in the modified phRLSV40 vector creating phRL_Stin2.9, phRL_Stin2.10 and phRL_Stin2.12. To generate the dual constructs, these initronic constructs were digested with *BamHI* and *HindIII*, to extract the Stin2 VNTR plus the renillin gene. pGL3p_long and pGL3p_short, were digested with *BamHI* and *HindIII* to extract the LPR VNTR, the SV40 promoter and the β-lactamase gene. These fragments were ligated together, creating the dual constructs: L9, L10, L12, S9, S10, and S12. Positive clones were confirmed by sequencing. The mammalian pCI-CTCF expression construct used has been described previously (Klenova *et al*., 2004).

**Reverse Transcription PCR**

Total RNA was extracted from rat prefrontal cortical cells with Trizol reagent (Invitrogen, UK). Contaminating genomic DNA was digested with DNase (Promega) at 37 °C for 30 min. 1 μg of total RNA was reverse-transcribed to single-stranded cDNA using Reverse Transcription System (Promega) and random primers according to the manufacturer’s instructions. PCR was carried out on cDNA to confirm *SLC6A4* mRNA expression using the primers forward: 5’-ttcctcctgtccgtcattgg-3’ and reverse 5’- ggtggatctgcaggacatgg-3’ from exons 1 and 4, respectively. PCR reaction mixes contained 50 ng of cDNA template, 0.2 μM of each primer, 2.5 units GoTaq DNA polymerase (Promega), 1x reverse transcriptase buffer (Promega), 0.2 mM dNTPs, 1.5 mM MgCl_2_, and water to a final volume of 20 μl per reaction. PCR was performed for 40 cycles: 94 °C denaturing (30 sec.), 50 °C annealing (30 sec.), 72 °C extension (45 sec.), using a Px2 thermal cycler (Thermo Scientific).

**Primary cell culture, transient transfections, and luciferase assays**

Male Wistar albino rats (2-7 days old) were used to generate primary cell cultures from cortical tissue. All animals were culled under local and national schedule one guidelines. All procedures were carried out according to the UK Home Office regulations. Briefly, cortices were collected in dissection solution HBSS (Invitrogen-Gibco). Tissue was dissociated enzymatically with trypsin-EDTA (Sigma-Aldrich Ltd.) and mechanically with fire-polished Pasteur pipettes. Cells were subsequently plated into poly-D-lysine (100 ng/ml) coated 24-well plates (5 x 10^5^ cells/well) containing medium I (DMEM, 10% FCS, 100 units/ml penicillin, 100 µg/ml streptomycin) for 7 hrs at 37 ^o^C/5% CO_2_. Medium was changed to Neurobasal-A containing 2% B27 supplement (Invitrogen-Gibco), 2 mM L-glutamine and 1 µg/ml of gentamycin (Invitrogen-Gibco), and cells were cultured overnight prior to transfection. Reporter constructs (1 µg) or modified phRLSV40 (1 µg) and modified pMLuc-2 (*Firefly* luciferase, for use as an internal control) were co-transfected into cultures using ExGen500 *in vitro* transfection reagent following manufacturer’s guidelines (Fermentas). For co-transfection with CTCF, cells were transfected with 1 µg of reporter gene and 1 µg of expression vector pCI-CTCF, or to standardise total DNA concentration, pGL3basic (Promega). Cells were harvested and assayed using the Dual Luciferase Reporter Assay System (Promega) and luminescence was measured using a Glomax 96 microplate luminometer (Promega). Mean and S.E.M were calculated from the results of three independent experiments performed in triplicate.

**Statistical analysis**

Data obtained from the reporter gene experiments were subject to statistical analysis using one-way ANOVA and a significance level of 0.05. The Dunnett (2-sided) t method was used as a *Post Hoc* test to account for multiple comparisons. The results corresponding to the comparisons between individual cells and groups were then reported and any significant result highlighted.

**Cell culture**

JAr cells (human placental choriocarcinoma [ATCC HTB-144; American Type Culture Collection, Manassas,VA]) were maintained as monolayers in RPMI-1640 medium (Bioclear, Autogen) supplemented with 10% heat-inactivated foetal calf serum (Hy-Clone, Logan, UT), 2 mg/ml glucose, 1 mM sodium pyruvate and penicillin/streptomycin (100 units/ml each).

**Chromatin Immunoprecipitation**

DNA and protein from 10^7^ JAr cells were cross-linked with 1% formaldehyde and mixed thoroughly at room temperature for 10 min. Chromatin immunoprecipitation (ChIP) was performed using ChIP-IT express (Active Motif) according to the manufacturers’ instructions. The antibodies used were as follows: monoclonal mouse anti-human CTCF (BD Transduction Laboratories) and polyclonal rabbit anti-mouse IgG which acted as a negative control (Abcam).

Briefly, cross-linking was quenched by addition of Glycine “Stop-Fix” buffer and nuclei were prepared and lysed in lysis buffer supplemented with protease inhibitor cocktail (PIC) and phenylmethylsulphonyl fluoride (PMSF) to ensure that the protein /DNA interactions were preserved during chromatin purification and immunoprecipitation. Chromatin was sheared by sonication, 10x 30 sec pulses at 50% power using a Microson sonicator (Misonix). Efficacy of sonication was determined by agarose gel electrophoresis. Following phenol-chloroform extraction, DNA concentration was determined. 6.3 μg of sheared chromatin, 3 μg of antibody and 25 μl of protein G magnetic beads were mixed for each immunoprecipitation and the samples were incubated at 4 °C overnight on a rotating wheel. Following this step the samples were transferred to clean siliconised tubes to reduce background contamination. DNA was eluted, cross-links were reversed and the DNA was purified before subsequent PCR analysis. The PCR conditions were 30 cycles consisting of 95 °C denaturing step (1 min), 65 °C annealing step (1 min), 72 °C extension step (1 min), in a Px2 thermal cycler (Thermo Scientific). Primers used for LPR amplification were forward: 5’- ggggtacccctggcgttgccgctctgaatgc-3’ and reverse 5’-ccgctcgagcggagggactgagctggacaaccac-3’.

**Electrophorectic mobility shift assays**

The human recombinant CTCF protein purified from baculovirus was obtained as described in Chernukhin *et al*., (2000). An oligonucleotide pair, 5’ tcgacccctcgcagtatcccccctgca 3’, (100 ng) designed as overhanging complementary strands, was annealed by denaturing at 100 °C for 5 mins, then by slow cooling to room temperature, followed by labelling with α-^32^P dATP (specific activity 6000 Ci/mM) (Amersham) using DNA Polymerase I, Large (Klenow) Fragment (NEB).

EMSA assays were performed in a 5 µl final volume of binding buffer containing 20 mM HEPES-pH 7.5, 20% glycerol, 0.2 mM EDTA, 0.25 mM DTT, 0.5 mM PMSF, 0.1 M KCl, and 5 µg of poly dI-dC (deoxyinosinic-deoxycytidylic acid) (Sigma). 1 µg purified CTCF was incubated with 1×10^4^ c.p.m. labelled oligonucleotide probe for 30 min at room temperature in the presence or absence of 10-100 fold molar excess of unlabeled homologous 5’-tcgacccctcgcagtatcccccctgca-3’ or Stin2 5’-tgcaggctgtgacctgggat-3’ competitor. The DNA–protein complexes were resolved on 5.5% nondenaturing polyacrylamide gel (19:1 crosslinking ratio), dried and exposed overnight to Kodak X-Omat autoradiography film.
